# Supplementary material for: A high-resolution mRNA expression time course of embryonic development in zebrafish
Source: eLife. 2017 Nov 16;6:e30860. doi: 10.7554/eLife.30860 (PMC5690287; doi:10.7554/eLife.30860)
Supplement: Supplementary file 6. [file elife-30860-supp6.zip › biolayout-clusters-files/Cluster078-genes.html]

Cluster078


# Cluster078: Genes

| Ensembl ID | Gene Name | Chr | Start | End | Biotype |
| --- | --- | --- | --- | --- | --- |
| ENSDARG00000030532 | ARHGEF12 (1 of many) | 15 | 22510542 | 22601634 | protein\_coding |
| ENSDARG00000008119 | ENSDARG00000008119 | 20 | 29773995 | 29780904 | protein\_coding |
| ENSDARG00000032237 | anapc4 | 20 | 23576281 | 23595612 | protein\_coding |
| ENSDARG00000039328 | ccndbp1 | 6 | 60048130 | 60054349 | protein\_coding |
| ENSDARG00000043011 | chst14 | 20 | 25840889 | 25849317 | protein\_coding |
| ENSDARG00000034056 | csnk1g2b | 8 | 20463804 | 20527870 | protein\_coding |
| ENSDARG00000008757 | entpd6 | 17 | 21082465 | 21103739 | protein\_coding |
| ENSDARG00000017242 | garem | 7 | 41060447 | 41068501 | protein\_coding |
| ENSDARG00000030267 | gtf3aa | 5 | 67302476 | 67315375 | protein\_coding |
| ENSDARG00000036076 | heatr3 | 7 | 37627363 | 37648217 | protein\_coding |
| ENSDARG00000013025 | ints6 | 9 | 24014571 | 24030835 | protein\_coding |
| ENSDARG00000076716 | palb2 | 1 | 8440888 | 8450167 | protein\_coding |
| ENSDARG00000097264 | si:ch211-40k21.5 | 24 | 36365183 | 36383309 | protein\_coding |
| ENSDARG00000058076 | snf8 | 12 | 20531083 | 20536411 | protein\_coding |
| ENSDARG00000060506 | sos2 | 13 | 36513123 | 36537526 | protein\_coding |
| ENSDARG00000091851 | tsen2 | 11 | 561519 | 570007 | protein\_coding |
